# “Young Care”: A Community-Based Intervention to Transform Youth Mindsets on Elder Care in Thailand—Program Development and Outcome Evaluation

**DOI:** 10.3390/ijerph22081206

**Published:** 2025-07-31

**Authors:** Ranee Wongkongdech, Darunee Puangpronpitag, Tharinee Srisaknok, Kukiat Tudpor, Niruwan Turnbull, Souksathaphone Chanthamath, Adisorn Wongkongdech

**Affiliations:** 1 Faculty of Medicine, Mahasarakham University, Mueang Maha Sarakham 44150, Maha Sarakham, Thailand; ranee.w@msu.ac.th (R.W.); pdarunee@gmail.com (D.P.); 2 International and National Collaborative Network and Innovation for Community Health Development Research Unit (INCNI-CHD), Mahasarakham University, Mueang Maha Sarakham 44150, Maha Sarakham, Thailand; tharinee.s@msu.ac.th (T.S.); schanthamath1987@gmail.com (S.C.); 3 Faculty of Pharmacy, Mahasarakham University, Mueang Maha Sarakham 44150, Maha Sarakham, Thailand; 4 Faculty of Public Health, Mahasarakham University, Mueang Maha Sarakham 44150, Maha Sarakham, Thailand; kukiat.t@msu.ac.th (K.T.); niruwan.o@msu.ac.th (N.T.); 5 Public Health and Environmental Policy in Southeast Asia Research Cluster (PHEP-SEA), Mahasarakham University, Mueang Maha Sarakham 44150, Maha Sarakham, Thailand; 6 Department of Medicine, Khammouane Provincial Hospital, Thakhek 9RQ4+VQ2, Laos

**Keywords:** youth caregiving, elder care, aging society, Thailand, community-based intervention

## Abstract

**Background:** Thailand is rapidly transitioning into an aging society, creating an intergenerational caregiving gap that strains existing support systems. **Objective:** This study evaluated the effectiveness of “Young Care,” a community-based intervention designed to enhance youth knowledge, attitudes, and caregiving practices (KAP) toward older adults. **Methods:** A two-day structured training was conducted in Maha Sarakham Province in 2023 using a pre-post mixed-methods design. Middle and high school students participated in lectures, multimedia sessions, and experiential learning activities related to caregiving. Quantitative data were collected using validated KAP questionnaires, while qualitative insights were obtained from focus group discussions involving students, older persons, caregivers, and local leaders. **Results:** Post-intervention analysis revealed significant improvements in knowledge and attitudes (*p* < 0.001), accompanied by increased empathy, caregiving initiative, and a sense of moral responsibility among participants. **Conclusions:** The initiative fostered formal partnerships among schools, local governments, healthcare providers, and universities through memoranda of understanding. These collaborations enabled budgetary support and outreach to out-of-school youth, positioning “Young Care” as a scalable, youth-centered strategy to address Thailand’s long-term care challenges.

## 1. Introduction

Thailand is among the fastest-aging countries in Southeast Asia, with projections indicating that over 30% of the population will be aged 60 years or older by 2035 [1]. This demographic shift presents significant challenges to the healthcare and social systems, particularly the increasing demand for long-term care (LTC) services [2,3]. Traditionally, caregiving responsibilities have been primarily borne by family members, particularly women, in rural and semi-urban communities [4]. However, declining fertility rates, urban migration, and economic pressures have weakened multigenerational support structures and eroded the foundation of informal caregiving [5,6].

In response to these challenges, Thailand enacted the Older Persons Act B.E. 2546 (2003), which aligns with the Madrid International Plan of Action on Ageing (2002), affirming the rights, dignity, and well-being of older persons [7,8]. Despite these efforts, family caregivers (FCGs) remain under-supported, often lacking formal training, institutional resources, and emotional support [9,10,11]. Many caregivers must balance caregiving responsibilities with work or schooling, resulting in caregiver stress, burnout, and inconsistent care quality [12,13,14].

The gap in caregiving capacity is particularly evident among adolescents and youth. A recent cross-sectional study of 1551 secondary school students in Northeastern Thailand found that over 85% had poor knowledge of elder care, especially in emergency response and medication safety. For instance, fewer than 10% could identify abnormal pulse rates or respond appropriately to diabetic crises. While the majority expressed respect for older family members, only 9.4% demonstrated highly positive attitudes toward caregiving, and more than one-third reported feelings of discomfort or indifference when assisting older adults in public settings [15].

These findings underscore the urgent need for early, culturally grounded interventions that empower adolescents with both knowledge and emotional readiness for caregiving to older adults. The “Young Care” initiative was developed to address this gap through a participatory, community-based approach tailored to diverse school settings. Drawing on intergenerational and experiential learning models, as well as people-centered principles of care across the life course [16], the program equips youth not only with practical eldercare skills but also with prosocial values, moral responsibility, and sustained engagement in caregiving [15,17,18]. This approach is also consistent with Kavanaugh et al. (2016) [19], who emphasized the importance of preparing youth as contributors to long-term care systems, particularly in aging societies. Pilot implementation further suggests that structured caregiving education can foster empathy, mitigate intergenerational stigma, and promote civic responsibility—findings that align with the existing literature on adolescent and intergenerational development [15,20,21,22,23].

## 2. Materials and Methods

### 2.1. Study Design

This study employed a mixed-methods intervention design structured into three sequential phases: (1) Understanding the Problem, (2) Designing Solutions and Program Implementation, and (3) Program Evaluation.

### 2.2. Study Setting and Participants

The study was conducted between 1 July and 30 September 2023, in Mahasarakham Province, Northeastern Thailand. Participant recruitment involved selecting three secondary schools to represent urban, rural, and semi-urban settings: Phadungnaree School (urban, with 3688 students), Mittraphap School in Kaedam District (small rural, 240 students), and Nachaueak Pittayasan School in Nachaueak District (large semi-urban, with 1945 students) [24]. Students in Grades 9–11 (Mathayom 3–5), aged 13–18 years, were invited to participate based on their interest and willingness to complete the entire program. Teachers assisted in screening for commitment and availability through interviews. Although the initial plan was to enroll 60 students proportionally across schools, high interest led to the inclusion of 71 participants. Of these, 60 completed both pre- and post-evaluations, yielding an attrition rate of 15.5%, which is within acceptable limits for school-based interventions.

### 2.3. Ethical Considerations

Permission to conduct the study was obtained from the Secondary Education Service Area Office (Mahasarakham), and all participating schools provided institutional support. Informed consent was obtained from both parents/guardians and students through the completion of signed consent forms. Ethical approval was granted by the Ethics Committee of Mahasarakham University (Ref. No. 151-110/2023).

### 2.4. Intervention Development and Implementation

A baseline KAP (Knowledge, Attitudes, Practices) survey was conducted among 1551 students to identify knowledge gaps and patterns of caregiving behavior. Focus groups with students and teachers further informed expectations and learning preferences. A participatory approach was adopted to co-design a culturally relevant five-module curriculum. The two-day intervention emphasized both knowledge acquisition and soft skills development, with training modules on the following: (1) Understanding Aging and Elder Rights, (2) Emotional Intelligence and Empathy, (3) Basic Caregiving Skills, (4) Communication Across Generations, and (5) Youth as Community Change Agents. A detailed training schedule is available in Supplementary Material File S2.

Following curriculum development, the program was reviewed by subject matter experts and piloted with a sample of target students for clarity and cultural appropriateness. The finalized intervention was delivered as a two-day intensive workshop involving lectures, hands-on caregiving tasks, and supervised home-based practice sessions. School staff coordinated logistics, including transportation and meals.

### 2.5. Data Collection and Analysis

Quantitative data were collected using a 35-item validated KAP questionnaire administered pre- and post-intervention. Data analysis was conducted using SPSS version 28.0. Paired *t*-tests assessed changes in KAP mean scores, while McNemar–Bowker tests evaluated categorical changes. Cronbach’s alpha demonstrated acceptable internal consistency: 0.78 (knowledge), 0.82 (attitudes), and 0.75 (practices). The complete questionnaire is provided as Supplementary Material File S1.

For the qualitative component, semi-structured interviews were conducted with key informants, including school principals and the Director of the Secondary Education Office. Focus group discussions included representatives from academic affairs, student affairs, guidance, school health, and health education. Data were analyzed thematically using content analysis, with stakeholder engagement throughout the coding and validation process.

### 2.6. Reflexivity and Researcher Positionality

The research team comprised individuals with expertise in both quantitative and qualitative research methodologies, accumulated through academic and professional experience in public health and education. This background provided a comprehensive understanding of the study context and the diverse perspectives of the participants.

During data collection, for the quantitative phase, coordination followed established inter-agency protocols to ensure standardized administration of questionnaires [12,13,24]. For the qualitative phase, the researchers maintained an awareness of their positionality, ensuring that interview questions and focus group discussions were open-ended and allowed participants to express their views freely. While our collective experience informed the initial framing of the research questions, efforts were continuously made to minimize the imposition of pre-conceived notions during interactions.

In data interpretation, quantitative analysis strictly adhered to statistical methods, ensuring objectivity and data-driven conclusions. For the qualitative component, the thematic analysis process involved rigorous engagement with stakeholders during coding and validation. This multi-perspectival approach aimed to enhance the trustworthiness of the interpretations by mitigating potential individual biases and grounding the findings firmly in the participants’ perspectives.

## 3. Results

### 3.1. Participant Characteristics

A total of 71 secondary school students, aged 13–18 years, participated in the Young Care training program. The mean age was 15.7 ± 1.4 years. Most participants were female (63.3%) and reported living with older adults in their households (84.2%).

### 3.2. Efficacy of the ‘Young Care’ Program by Development Phase

To comprehensively present the impact of the Young Care initiative, outcomes are structured by the three phases of the research and development (R&D) process, integrating both quantitative improvements and qualitative narratives.

- Phase 1—Understanding the Problem

A baseline KAP survey of 1551 secondary school students across four Northeastern provinces revealed significant gaps in caregiving readiness. Most students had poor knowledge, held only moderately positive attitudes, and had limited hands-on experience with elder care. Although many appreciated the value of older adults in family life, few were confident or willing to assist with practical caregiving tasks. These findings underscore the need for targeted intervention to build caregiving competencies among youth [1].

Key Insight: These findings underscore the importance of cultivating both mindset shifts and caregiving skills among adolescents.

- Phase 2—Curriculum Design and Implementation

The participatory development process yielded a culturally relevant five-module curriculum that was well-received by both students and educators. Feedback from pilot testing and expert reviews highlighted the curriculum’s clarity, age-appropriateness, and practical relevance. Teachers reported high engagement, and students noted that simulations and real-life caregiving tasks helped build their confidence and caregiving skills.

Although the original target was 60 students, participation increased to 71 due to strong interest, particularly from Phadungnaree School located near the training site. The Faculty of Medicine at Mahasarakham University provided additional support to accommodate the expanded group.

Key Insight: Strong community engagement and the relevance of content led to high participation and enthusiasm, suggesting that youth are highly receptive to structured caregiving education when it is aligned with their social context [11].

- Phase 3—Program Evaluation

The program’s efficacy was assessed through quantitative pre- and post-intervention KAP comparisons, as well as qualitative reflections gathered through interviews, focus groups, and participatory observation.

Table 1 presents the overall KAP scores before and after the intervention. Substantial gains were observed in knowledge (Mean difference = 3.02, *p* < 0.001) and attitudes (Mean difference = 4.15, *p* < 0.001), with caregiving practices also showing marked improvement (*p* < 0.001). Effect size analysis indicated a large effect for knowledge (Cohen’s *d* = 0.94) and a moderate-to-large effect for attitudes (Cohen’s *d* = 0.72), suggesting a meaningful impact of the program.

Table 2 summarizes the paired *t*-test results comparing pre- and post-intervention scores, confirming statistically significant improvements in knowledge, attitudes, and caregiving practices (all *p* < 0.001). Knowledge scores increased by an average of 3.02 points (Cohen’s *d* = 0.94), indicating a large effect size. Attitudes improved by 4.15 points (Cohen’s *d* = 0.72), reflecting a moderate-to-large effect. Practice scores also rose by 5.3 points, suggesting meaningful behavioral change, although the effect size was not calculated. These results highlight the program’s effectiveness in fostering both cognitive and behavioral growth among participants.

#### 3.2.1. Qualitative Results

Semi-structured interviews and focus group discussions revealed emotional and behavioral impacts on students and families. Students exhibited greater initiative and empathy post-training. Caregivers reported that youth became more engaged and proactive in household elder care. Statements such as “My granddaughter now checks on me every day” and “He offers to help without being told” exemplify meaningful changes.

Community caregivers and older adults expressed appreciation for the students’ efforts, often describing feelings of moral fulfillment and hope. Teachers and local leaders observed an increase in student leadership and interest in caregiving.

Key Insight: The program not only enhanced measurable caregiving competencies but also fostered deeper emotional connections, reinforcing caregiving as a valued youth role in both family and community settings [25].

#### 3.2.2. Sustainability and System-Level Impact

- Two schools formed Young Care clubs with initial funding of THB 3000 each to promote continued caregiving activities.
- A LINE group named “Young Carer” was established, eventually expanding to 110 active members. While the original cohort included 71 trained students, the additional members comprised peers who were inspired by the program through school assemblies, peer-sharing, and youth-led community outreach, demonstrating the program’s capacity for diffusion beyond direct participants.
- The program was integrated into school assemblies and leadership development activities.
- Local education authorities expressed interest by requesting extended training sessions for broader student groups, reflecting early policy engagement.

These developments suggest that the Young Care program not only improved measurable KAP outcomes but also catalyzed emotional growth, peer networking, and community-driven caregiving efforts. Its spillover effect and sustained student-led activities illustrate its potential as a scalable and socially embedded model for youth caregiving education [26,27].

## 4. Discussion

This study demonstrates that the “Young Care” program effectively promoted youth engagement in elder care through both cognitive and emotional pathways. Statistically significant improvements in Knowledge, Attitudes, and Practices (KAP) reflect the program’s ability to address key gaps in caregiving readiness among adolescents—particularly in a rapidly aging society like Thailand [15,28]. These findings align with global policy recommendations from the World Health Organization, particularly the Decade of Healthy Ageing 2020–2030, which emphasizes life-course approaches, intergenerational solidarity, and the inclusion of youth in building sustainable, age-friendly communities [29].

While all domains showed positive changes, improvements in attitudes were relatively modest compared to those in knowledge and practice. This is consistent with the Theory of Planned Behavior, which suggests that deeply rooted beliefs and social norms shape attitudes and require sustained exposure to bring about meaningful change [30]. Hence, although a two- or three-day intervention can rapidly improve factual knowledge and caregiving skills, longer-term interventions may be necessary to foster enduring attitude change [22,25].

Qualitative findings revealed more profound shifts beyond knowledge acquisition. Many students progressed from passive observers to active caregivers, demonstrating empathy, moral responsibility, and initiative. Students’ reflections, such as “He now offers to help cook without being told,” exemplify how the program fostered meaningful transformation. These outcomes align with Adolescent Development Theory, which posits that opportunities for meaningful contribution promote prosocial behavior, self-efficacy, and social competence [23,31].

From the perspective of older adults and caregivers, the program delivered psychosocial value. Elders reported feeling “hopeful” and “emotionally connected,” describing students’ care as heartfelt and consistent. Such narratives align with evidence from intergenerational programs, which show positive effects on elder well-being, particularly in aging societies with rising social isolation [2,22,32,33].

The school- and community-based implementation model also contributed to sustainability. Supportive mechanisms—such as student-led Young Care clubs, peer networks (e.g., the LINE group of 110 youth caregivers), and school budget allocations—reflect strong community ownership. These elements are essential for program continuity, especially in semi-urban and rural areas where formal caregiving systems remain limited [2,29,34].

Furthermore, caregiving expectations in Thai culture have traditionally been shaped by gendered socialization, with daughters and granddaughters often expected to care for older adults [29]. The Young Care program challenged this norm by encouraging inclusive participation and empowering male students to take equal responsibility. By promoting caregiving as a shared duty, the program helped shift gendered perceptions and supported evolving attitudes toward equity in elder care, in line with national youth development trends [30]. These gender-transformative elements are essential for building inclusive and sustainable care systems.

This model reinforces the findings of Wongkongdech et al., who emphasize that early caregiving exposure not only builds competence but may also inspire future careers in health and social care. By engaging youth at an early stage, the program helps redistribute care burdens in families, supports aging-in-place models, and strengthens community resilience.

Moreover, this intervention makes a meaningful contribution to Thailand’s national discourse on long-term care. Given demographic trends, declining family size, and digital exclusion among older adults, programs like Young Care offer practical, culturally embedded solutions that bridge generational divides while enhancing caregiving capacity [15,29]. The success of this initiative highlights the crucial role of youth as key actors in addressing population aging—not just as future professionals, but also as present-day caregivers within their households and communities [17,28].

### 4.1. Policy Implications

This initiative demonstrates a scalable, community-based model for integrating elder care education into Thailand’s national moral and civic curriculum. Educational authorities and local governments should consider supporting similar interventions as a strategic response to the growing caregiving gap in aging societies [6,7]. Investment in youth-led caregiving programs can help alleviate the burden on formal healthcare services, particularly in rural and semi-urban areas where resources are often limited [10,27].

Furthermore, the Young Care initiative aligns with both national and international policy frameworks. Domestically, it supports the objectives outlined in Thailand’s Elderly Act B.E. 2546 (2003), which emphasizes the roles of family and community in caring for and protecting older persons [7]. Internationally, it echoes the principles of the Madrid International Plan of Action on Ageing (2002), particularly the call for increased youth involvement and intergenerational solidarity in addressing population aging. By engaging adolescents in caregiving roles, the program reinforces the life-course approach and contributes to building age-inclusive communities, aligning with both frameworks [8,16,19].

These findings align with global policy recommendations from the World Health Organization, which emphasize the importance of life-course approaches and intergenerational engagement in strategies for active and healthy aging [29,35]. Similarly, UNICEF emphasizes the importance of empowering adolescents as agents of social change, particularly in contexts of demographic transition and shifting family structures [16,36].

The model’s alignment with Thailand’s National Strategy on Aging (2018–2037) and decentralized health governance enhances its feasibility, allowing for localized adaptation and ownership. The active involvement of school administrators, caregivers, and older adults reflects strong community buy-in and offers a foundation for long-term sustainability [7].

Notably, school leaders have proposed incorporating the Young Care program into a formal “credit bank” system, allowing students to earn academic or vocational credits for their participation in caregiving. Such an initiative would institutionalize youth contributions, strengthen their future educational and career opportunities, and reinforce caregiving as a valued civic responsibility [33]. Furthermore, this model promotes intergenerational solidarity and aligns with Thailand’s policy vision of inclusive, participatory aging [7,37]. It may also serve as a prototype for similar youth-led caregiving initiatives across Southeast Asia, where aging populations and evolving family dynamics present shared regional challenges [1,17].

### 4.2. Limitations

This study is limited by its relatively small sample size and the short-term follow-up period. The program was implemented in a single province, which may limit generalizability. Additionally, the self-reported nature of the survey may introduce social desirability bias [12]. Future research should consider larger, randomized trials with long-term impact evaluations and the inclusion of objective caregiving performance metrics [12].

## 5. Conclusions

The Young Care program demonstrates the feasibility and efficacy of youth-centered interventions in enhancing caregiving readiness, moral responsibility, and intergenerational empathy. Beyond improving students’ knowledge and attitudes, the initiative fostered deeper connections between adolescents, older adults, and community caregivers [25].

Embedding caregiving education within civic and moral curricula—supported by schools, local authorities, and families—offers a proactive approach to preparing Thai youth for the realities of an aging society [20,33]. Institutional mechanisms such as credit banking can further formalize and incentivize youth participation, paving the way for scalable, community-driven elder care solutions [37].

## Figures and Tables

**Table 1:** Comparison of Knowledge, Attitudes, and Practices (KAP) scores before and after the intervention (n = 60).

Domain	Pre-Test (Mean ± SD)	Post-Test (Mean ± SD)	*p*-Value
Knowledge	13.65 ± 3.36	16.67 ± 3.00	<0.001
Attitudes	51.15 ± 8.41	55.30 ± 8.46	<0.001
Practices	21.4 ± 3.6	26.7 ± 3.2	<0.001

Effect sizes indicated large gains for knowledge (Cohen’s *d* = 0.94) and moderate-to-large gains for attitude (Cohen’s *d* = 0.72).

**Table 2:** Paired *t*-test results of Knowledge and Attitude scores before and after the “Young Care” intervention (n = 60).

Variable	Time Point	n	$\bar{X}$	SD	Mean Difference	*t*	95%CI	*p*-Value *
Knowledge of Elder Care	Before	60	3.77	2.14	−1.09	−30.16	−1.17, −1.02	<0.001
	After	60	9.87	3.64				
Attitudes Toward Elder Care	Before	60	3.39	0.52	−0.82	−8.46	−1.01, 0.62	<0.001
	After	60	3.88	0.47				

* Note: All *p*-values are <0.001, indicating statistically significant differences.

## Data Availability

Due to ethical concerns and privacy protections for study participants, the data are not publicly available.

## Supplementary Materials

The following supporting information can be downloaded at: https://www.mdpi.com/article/10.3390/ijerph22081206/s1, Supplementary Material File S1: KAP Questionnaire on Elder Care; Supplementary Material File S2: Training Schedule; Workshop Title: “Young Carer Capacity Building.
