# Supplementary material for: “Young Care”: A Community-Based Intervention to Transform Youth Mindsets on Elder Care in Thailand—Program Development and Outcome Evaluation
Source: Int J Environ Res Public Health. 2025 Jul 31;22(8):1206. doi: 10.3390/ijerph22081206 (PMC12386511; doi:10.3390/ijerph22081206)
Supplement: Supplementary file 1 [file ijerph-22-01206-s001.zip › ijerph-3742750-Supplementary_Material_1_KAP_Questionnaire.pdf]

Supplementary Material 1: KAP Questionnaire on Elder Care

This questionnaire was developed to assess adolescents' Knowledge, Attitudes, and Practices (KAP) regarding elder care. It consists of three main sections. The tool was validated and pilot-tested prior to implementation.

Part 1: Practices

In the past year, have you helped or performed the following activities for older persons? Please mark the frequency.

Activities	1–2 times/year	1–2 times/month	1–2 times/week	3–5 times/week	Everyday
1. Massage					
2. Dressing the elder					
3. Preparing the bed					
4. Assisting in the bathroom					
5. Feeding					
6. Conversing/social interaction					
7. Preparing medications					
8. Preparing meals					
9. Cooking meals					
10. Wound care					
11. Running errands (e.g., buying items, paying bills)					
12. Washing/ironing clothes					
13. Cleaning the room/house					
14. Washing dishes					
15. Buying snacks/gifts					
16. Assisting with physical activity (e.g., walking or bed exercises)					
17. Reading/watching TV together					
18. Sleeping beside the elder					
19. Others (please specify): ________					

Part 2: Knowledge about Elder and Dependent Care

Instructions: Please mark an X in the column that best matches your answer to each question.

No.	Statement	Yes	No	Not Sure
1.	Assessment of daily living abilities helps identify problems and plan appropriate care.			
2.	An abnormal body temperature to watch for is a temperature of≥39.0°C; the axillary temperature is higher than the oral temperature.			
3.	Sponging a fevered person involves wiping from extremities toward the heart and applying a compress to the armpits.			
4.	Abnormal pulse rate is >100 or <60 beats per minute.			
5.	Normal blood pressure is around 120/80 mmHg; slightly higher values are acceptable in the elderly.			
6.	Blood pressure readings are the same regardless of position or time of measurement.			
7.	Antibiotics can be stopped once symptoms improve.			
8.	Elderly individuals who are bedridden should maintain a consistent position to prevent falls or injuries.			
9.	Key care for feeding bedridden elders includes preventing choking and ensuring food hygiene.			
10.	People with diabetes should reduce or avoid sweets; however, fruit is unlimited, provided it is not overly sweet.			
11.	All post-meal medications must be taken immediately after or with food.			
12.	Expired medications should only be assessed based on the printed expiration date.			
13.	Herbal or dietary supplements are safe and can be consumed without restriction.			
14.	A sprained ankle should be treated immediately with heat compress or warming ointment.			
15.	If an elder fall in the bathroom or bed, the first action is to wake and lift them.			
16.	If food or foreign objects are aspirated, call 1669 and wait for emergency services to arrive.			
17.	Diabetic unconsciousness is always due to low blood sugar.			
18.	If an elder with diabetes and hypertension has chest pain radiating to the arm, let them rest and observe.			
19.	Severe drug allergy symptoms include chest tightness and shortness of breath.			
20.	Facial droop or limb weakness may indicate stroke from blocked or ruptured cerebral vessels.			

Part 3: Attitudes Toward the Elderly and Elder Care

Instructions: Please mark the box that best represents your opinion on each statement regarding older persons and their care in the family or society.

- Strongly Agree = You fully agree with the statement.
- Agree = You agree with the statement for the most part.
- Not Sure = You are uncertain or only partially agree.
- Disagree = You disagree with the statement for the most part.
- Strongly Disagree = You fully disagree with the statement.

No.	Statement	Strongly Agree	Agree	Not Sure	Disagree / Strongly Disagree
1	Older persons are valuable to families and society.				
2	Older persons, especially those who are ill, are a burden to families in terms of time and cost.				
3	Caring for the elderly, even if not relatives, is virtuous and beneficial to oneself and one's family.				
4	Having older family members brings warmth and unity.				
5	The experiences of older persons help in solving problems.				
6	Older persons are often perceived as fussy, forgetful, and annoying.				
7	Older persons often talk about the past, which can be frustrating and tedious.				
8	I feel comfortable talking with older people.				
9	I can talk or consult with older persons about almost anything.				
10	Older persons walk and work slowly and are not efficient.				
11	Older persons should stay home and avoid outdoor activities to minimize the risk of danger.				
12	I feel embarrassed when taking care of older persons in public.				
13	Older persons may struggle to contribute to society due to outdated ideas and physical decline.				
14	I feel indifferent when I see news about older persons being abandoned or left alone.				
15	Caring for older persons is pointless because their bodies are deteriorating.				
16	I do not want to care for older persons; even minor tasks feel like a waste of time.				
17	Even basic care for older persons requires proper training.				

No.	Statement	Strongly Agree	Agree	Not Sure	Disagree / Strongly Disagree
18	Elderly care should address the physical, mental, emotional, and social needs of individuals in a holistic manner.				
19	Elderly persons should reside in nursing homes so that family members can work without interruption.				
20	Older persons should have the opportunity to spend their final days with family.				
21	If older persons speak incorrectly or in an unpleasant manner, they should be corrected until they are understood.				
22	Caring for older persons should only be done by adults or professionals.				
23	I am unable to care for older persons.				
24	Other				

- Authors: Ranee Wongkongdech, Darunee Puangpronpitag, Tharinee Srisaknok, Kukiat Tudpor, Niruwan Turnbull, Souksathaphone Chanthamath, Adisorn Wongkongdech
- Note: This 35-item questionnaire was developed by the research team to assess adolescents' knowledge, attitudes, and practices (KAP) regarding elder care in Thailand. The tool was reviewed by experts, pilot-tested among a similar population, and used in a pre-post intervention study. The items are grouped into three parts: Practices (Part 1), Knowledge (Part 2), and Attitudes (Part 3).
- This English version was translated from the original Thai version by the study team and verified by bilingual public health experts for clarity and conceptual equivalence.
