# Supplementary material for: “Young Care”: A Community-Based Intervention to Transform Youth Mindsets on Elder Care in Thailand—Program Development and Outcome Evaluation
Source: Int J Environ Res Public Health. 2025 Jul 31;22(8):1206. doi: 10.3390/ijerph22081206 (PMC12386511; doi:10.3390/ijerph22081206)
Supplement: Supplementary file 1 [file ijerph-22-01206-s001.zip › ijerph-3742750-Supplementary_Material_2_Training_Schedule.pdf]

Supplementary Material 2: Training Schedule

Workshop Title: “**Young Carer Capacity Building**”

Day 1

Time	Activities
08:30–09:00	- Pre-training assessment on knowledge, attitude, and practices related to elder care<br>- Ice-breaking and Team-Building Activities
09:00–12:00	- Participation agreement activities<br>- Situation analysis on aging society and its impact through:<br>• Short film (“Misdelivered Letter”)<br>• Group reflection: “Why should young people help elders live longer?”<br>• Group brainstorming on aging issues at various levels
12:00–13:00	- Lunch break
13:00–16:00	- Interactive session on communication skills and health assessments:<br>• History taking, vital signs<br>• Common health problems and management strategies<br>• Aging simulation using kits
16:00–16:30	- Summary discussion and reflection

Day 2

Time	Activities
08:30–09:00	Inspirational session
09:00–12:00	Skill Stations:<br>• Station 1: Health assessment and medication use<br>• Station 2: Positioning and physical rehabilitation<br>• Station 3: Thai traditional massage<br>• Station 4: Emergency care and referral preparation
12:00–13:00	Lunch break
13:00–16:00	Practical group work:<br>• Case interview and care planning<br>• Post-training evaluation<br>• Panel: “Young Care Club” and sustainability plans
16:00–16:30	Reflection session: “Ask Yourself: Are You Ready to Move Forward?”

Prepared and designed by:
Dr. Ranee Wongkongdech – Associate Professor, Mahasarakham University
Dr. Adisorn Wongkongdech – Associate Professor, Mahasarakham University
Dr. Tharinee Srisaknok – Lecturer, Mahasarakham University
Dr. Narongsak Chantawang – Associate Professor,

Dr. Walapa Tritip – Physical Therapist, Mahasarakham University
Mr. Maitree Thonsao – Emergency Medical Lecturer
